# Vitamin D_3_ Metabolism and Its Role in Temporomandibular Joint Osteoarthritis and Autoimmune Thyroid Diseases

**DOI:** 10.3390/ijms24044080

**Published:** 2023-02-17

**Authors:** Michał Szulc, Renata Świątkowska-Stodulska, Elżbieta Pawłowska, Marcin Derwich

**Affiliations:** 1 Department of Endocrinology and Internal Medicine, Faculty of Medicine, Medical University of Gdańsk, 80-952 Gdańsk, Poland; 2 Department of Pediatric Dentistry, Medical University of Lodz, 90-419 Łódź, Poland

**Keywords:** vitamin D_3_, bone turnover, degenerative joint disease, temporomandibular disorders, temporomandibular joint osteoarthritis, autoimmune thyroid diseases

## Abstract

The aim of this review was to present the metabolism of vitamin D_3_, as well as to discuss the role of vitamin D_3_ in bone metabolism, temporomandibular joint osteoarthritis (TMJ OA), and autoimmune thyroid diseases (AITD) on the basis of the literature. Vitamin D_3_ plays a significant role in human health, as it affects the calcium-phosphate balance and regulates the bone metabolism. Calcitriol impresses the pleiotropic effect on human biology and metabolism. Its modulative function upon the immune system is based on the reduction of Th1 cell activity and increased immunotolerance. Vitamin D_3_ deficiency may lead to an imbalance in the relationship between Th1/Th17 and Th2, Th17/Th reg, and is considered by some authors as one of the possible backgrounds of autoimmune thyroid diseases (AITD), e.g., Hashimoto’s thyroiditis or Graves’ disease. Moreover, vitamin D_3_, through its direct and indirect influence on bones and joints, may also play an important role in the development and progression of degenerative joint diseases, including temporomandibular joint osteoarthritis. Further randomized, double blind studies are needed to unequivocally confirm the relationship between vitamin D_3_ and abovementioned diseases and to answer the question concerning whether vitamin D_3_ supplementation may be used in the prevention and/or treatment of either AITD or OA diseases.

## 1. Introduction

Calcium ions play a significant role in several vital processes in the human body, including blood clotting, muscle contraction, proper nerve activity, and bone turnover, as well as affect the cell membrane activity [1]. The physiologic concentration of calcium within serum depends on calcium absorption from the gastrointestinal tract, the intensity of calcification processes, and finally on the urine calcium excretion. Calcium homeostasis is controlled by three hormones: parathyroid hormone (PTH), calcitonin, and 1,25-dihydroxycholecalciferol, also known as 1,25-dihydroxyvitamin D_3_ [1].

Vitamin D_3_ is a fat-soluble steroid prohormone, which is well-known in medicine for over 100 years, especially for its invaluable role in rickets prevention [2], maintenance of calcium-phosphate balance, and finally due to its role in bone metabolism [3]. The disorders of calcium metabolism are most commonly related to the imbalance of osteoblasts and osteoclast activity, leading to osteopenia and osteoporosis [4,5,6]. Moreover, deficits of vitamin D_3_ have also been found to affect the skin issues, autoimmune disorders, cancers, cardiovascular, and metabolic diseases [7,8]. In addition, 1,25-dihydroxyvitamin D_3_ increases the concentration of calcium ions within the extracellular fluid by increasing the renal calcium and phosphate reabsorption, by increasing the intestinal calcium absorption, as well as by increasing bone resorption [9].

Vitamin D_3_ is in great interest of many different specialties, including among others endocrinology, and dentistry. Low levels of vitamin D_3_ have been linked with the presence of recurrent aphthous stomatitis [10] and may increase in the risk of the development of periodontal disease [11].

Vitamin D_3_ presents immunomodulative properties, and therefore it may affect autoimmune thyroid diseases (AITD), including Hashimoto thyroiditis (HT) and Graves’ disease (GD) [12,13]. AITDs are considered as one of the most common autoimmune diseases, affecting 5% of the population [14]. HT is a form of chronic, lymphocytic inflammation. Auto-antigens, e.g., thyroid peroxidase or thyroglobulin, are responsible for the immunization and activation auto-reactive T cells, recruitment of B cells, and auto-antibody production. This chronic inflammatory process leads to disfunction of the thyroid gland with hypothyroidism [15]. Contrary to HT, GD is a thyroid lymphocytic inflammatory process ongoing with hyperthyroidism. Activated Treg and B cells produce TSH receptor stimulating autoantibodies, resulting in uncontrolled and excessive thyroid hormone production and subsequent growth of thyroid tissue [16].

Low serum levels of vitamin D_3_ may increase the risk of the development of autoimmune thyroid diseases [12]. Moreover, it has also been reported that autoimmune thyroid diseases affect the bone metabolism [17,18].

There has been found a correlation between the low level of 25-hydroxycholecalciferol and both the presence and the intensity of many rheumatic diseases, including rheumatoid arthritis (RA), systemic lupus erythematosus (SLE), spondyloarthropathies (SpA), and osteoarthritis (OA) [19]. Furthermore, the polymorphisms of vitamin D receptor gene appeared to be related to the osteoarthritis susceptibility in the spine [20].

OA is one of the most common joint diseases, which encompasses: articular cartilage, synovium, subchondral bone, adjacent muscles, as well as ligaments [21]. Several different factors which may lead to OA have been listed. Those are: mechanical, metabolic, and inflammatory ones [21]. Some authors have found the presence of the correlation between the low serum levels of 25-hydroxyvitamin D_3_ and knee OA both in younger and older patients [22,23]. However, low serum levels of 25-hydroxyvitamin D_3_ did not correlate with the clinical and radiological symptoms indicating the severity of knee OA [23].

Degenerative joint disease (DJD), including OA, is also the most common disease of the temporomandibular joints (TMJs). The most common symptom of TMJ OA is pain [24]. DJD is also accompanied by below listed radiographic findings, namely: pseudocysts, erosion of the articular surface, osteophytes, generalized sclerosis, subcortical sclerosis, and articular surface flattening [25].

As far as the serum levels of vitamin D_3_ are correlated with the presence of knee OA, it may be speculated that vitamin D_3_ also affects the presence of TMJ OA. If so, especially very low levels of vitamin D_3_ in serum might be one of the TMJ OA risk factors. So far it has been found that polymorphisms of vitamin D3 receptor may be correlated with the development of the temporomandibular disorders (TMD) [26]. Additionally, due to the fact that vitamin D_3_ may affect autoimmune thyroid diseases, which consequently are able to affect bone metabolism, it may be also speculated that there exists a relationship between autoimmune thyroid diseases and the development of TMJ OA.

Therefore, the aim of this review was to present the metabolism of vitamin D_3_, as well as to discuss the role of vitamin D_3_ in bone metabolism, TMJ OA, and autoimmune thyroid diseases on the basis of the literature.

## 2. Vitamin D_3_—Biochemical Structure and Metabolism

**Figure 1:**
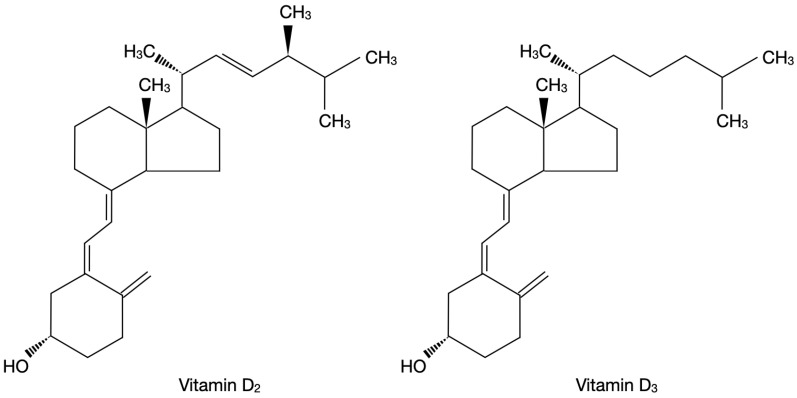
Chemical structure of vitamin D_2_ and vitamin D_3_ on the basis of the literature [27].

Vitamin D is a group of different steroid forms which encompasses among others vitamin D_2_ (ergocalciferol) and vitamin D_3_ (cholecalciferol) [27]. Figure 1 presents the chemical structure of vitamin D_2_ and vitamin D_3_ on the basis of the literature [27].

Vitamin D_3_ is a pro-hormone, but it was initially categorized as a vitamin, because it can be absorbed from dietary products, mainly oily fish, fish oil, dairy products, meat, eggs and some mushrooms. Nevertheless, the main source of vitamin D_3_ in humans is biosynthesis from 7-dehydrocholesterol in the course of photochemical reaction within the human skin [28].

Cholecalciferol is a prohormone that having been biosynthesized within the skin must undergo further transformations to become a biologically active hormone. Cholecalciferol becomes hydroxylated in the position of C25 by the enzyme 25-hydroxylase (CYP2R1, cytochrome P450 family) in the endoplasmic reticulum of the hepatocytes, or by CYP27A1 in the hepatic mitochondria. The product of the above described reaction is 25-hydroxyvitamin D_3_ (25(OH)D_3_) [29,30]. Enzymatic activity is regulated by negative feedback, where increased blood serum concentration of 25(OH)D_3_ decreases hydroxylation of vitamin D_3_. An excessive amount of vitamin D_3_ is stored in liver, muscles, or adipocytes [31]. 25-hydroxyvitamin-D_3_ is the major form of vitamin D_3_ circulating in the bloodstream with a long half-life time of 2–3 weeks. Therefore, measurement of blood serum concentration of 25(OH)D_3_ is a golden standard in the assessment of vitamin D3 deficits in humans [27].

25(OH)D_3_ is subsequently bound to the vitamin-D_3_ binding protein (DBP) and transported to the kidneys. Human DBP, also called group-specific component (GC), is a protein composed of 458 amino acids [32]. Most of the total plasma 25(OH)D_3_ is transported being bound to DBP, and only less than 10% is carried by the albumins [33]. The affinity of DBP is 10–100 times greater for 25(OH)D_3_ than to 1,25(OH)_2_D_3_ [34]. 

Subsequently, mainly in renal mitochondria, 25(OH)D_3_ becomes hydroxylated at the C1α position by the 1α-hydroxylase (CYP27B1) to the biologically active hormone 1,25(OH)_2_D_3_ (calcitriol). Enzyme CYP27B1 has also been found in other tissues, including skin, placenta, and many cells of the immune system that are able to produce calcitriol locally on tissue demand [35]. Renal 1α-hydroxylase activity is directly controlled (stimulated) by PTH, and inhibited by calcium, phosphates, or fibroblast growth factor 23 (FGF-23). Contrary to this, local biosynthesis of 1,25(OH)_2_D_3_ in peripheral tissues (e.g., by monocytes and macrophages) is controlled among others by the activity of the immune system, implying auto- and paracrine properties of vitamin D (apart from calcium and phosphate metabolism) [36]. 

The process of vitamin D_3_ inactivation, both 1,25(OH)_2_D_3_ and 25(OH)D_3_, starts with C24 hydroxylation by 24-hydroxylase (CYP24A1), mainly in kidneys, intestine, and bones. Further oxidation ends with calcitroic acid, water soluble molecules excreted to the bile [27].

**Figure 2:**
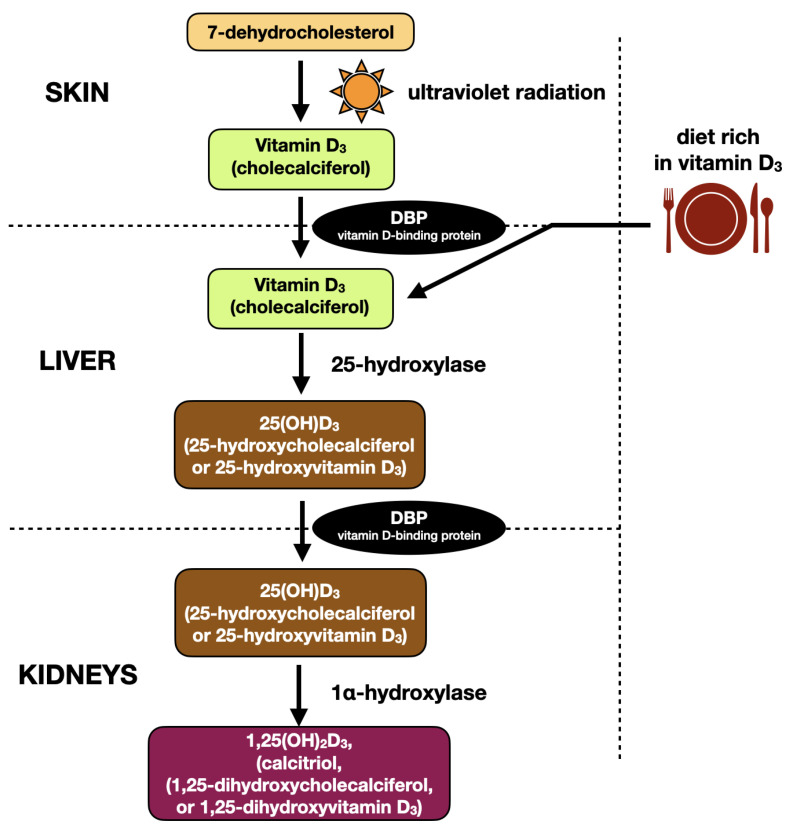
Metabolic pathway of vitamin D_3_ on the basis of the literature [27,31].

Figure 2 presents the metabolic pathway of vitamin D_3_ on the basis of the literature [27,31].

## 3. Vitamin D_3_ Receptor and Mechanisms of Action

Two different mechanisms describing biological activity of vitamin D_3_ have been discovered, namely: genomic (via vitamin D receptor) and non-genomic (pleiotropic effect) [37]. 

**Figure 3:**
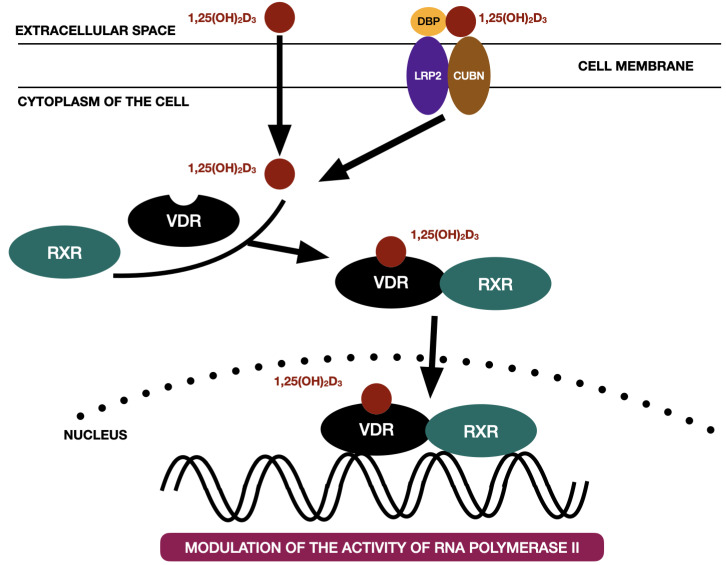
The classic vitamin D_3_ pathway on the basis of the literature [38]. 1,25(OH)_2_D_3_—calcitriol, CUBN—cubilin, DBP—vitamin D binding protein, LRP2—LDL receptor-related protein 2, RXR—retinoid X receptor, VDR—vitamin D receptor.

Vitamin D_3_ enters the cytoplasm of the cell either as a free molecule or in the process of endocytosis supported by the LDL receptor-related protein 2 (LRP2)—cubilin (CUBN) complex [38]. Within the genomic pathway, vitamin D3 binds to the vitamin-D receptor (VDR) and therefore forms the vitamin D3—VDR complex. VDR belongs to the steroid hormone nuclear receptors family, which includes: glucocorticoid, mineralocorticoid, estrogen, androgen, progesterone receptors [38]. VDR is a gene transcription factor consisting of three domains: C-terminal ligand-bind domain, N-terminal DNA-binding domain with two zinc fingers to link up with accessible DNA sites, and a clip area that binds these two together [39,40]. The VDR is activated by binding to calcitriol (1,25(OH)_2_D_3_) and subsequently transforms into heterodimer with retinoid receptor X (RXR). The calcitriol-VDR-RXR complex binds to the specific gene promoter region of the DNA and therefore is able to either promote or inhibit the RNA polymerase II specific VDR-dependent genes [10]. Figure 3 presents the classic vitamin D_3_ pathway on the basis of the literature [38].

1,25(OH)_2_D_3_ regulates more than 1000 genes in more than 200 tissues and cells within the human body [38,41]. In an in vitro experiment, when a cell culture model was exposed to a calcitriol concentration higher than physiologic (10–100 nM 1,25(OH)_2_D_3_), it took few hours to observe first biological effects [42].

The so-called non-genomic mechanism of action takes seconds to minutes and does not depend on VDR activation, nor gene transcription [38]. 1,25(OH)_2_D_3_ can cause calcium influx in cells. In a study from 1990 on osteogenic sarcoma cell line ROS 17/2.8, there was observed a rapid calcium inflow in cell culture treated with 1,25(OH)_2_D_3_ [43]. Another example of rapid, hormonally stimulated transport of calcium by enterocytes is called transcalathia [44]. Furthermore, calcitriol enables rapid (1–10 min) tissue uptake of calcium in myocardial chicken cells. Hormonal stimulations lead to microsomal membrane protein phosphorylation and activation of cyclic-AMP pathway [45]. According to the free hormone hypothesis, vitamin D_3_ is a hydrophobic molecule, which is able to naturally enter the phospholipid membrane, thus probably no membrane receptor or specific protein is needed in transmembrane transport [46]. Therefore, various pathways are known to be regulated by vitamin D_3_. This creates a possibility for clinical practice to individualize cell-specified therapy by vitamin D_3_ and its analogues.

## 4. Vitamin D_3_ and Immune System

Various immune cells express VDR, thus active vitamin D_3_ plays a vital role in human immune system. The VDR is present in both B and T lymphocytes, as well as in antigen presenting cells (APC), including monocytes, macrophages and dendritic cells [47]. Interestingly, leukocytes, especially APCs, are also able to activate circulating 25(OH)D_3_ to 1,25(OH)_2_D_3_ through 1α -hydroxylase (CYP27B1) [48]. Biological effects of calcitriol on leukocytes affect: cell proliferation, differentiation, maturation, and apoptosis (programmed cell death). Furthermore, 1,25(OH)_2_D_3_ affects the immunological balance between cell-mediated (Th1) and humoral (Th2) response [49].

The antigen presenting cells specialize in presenting hostile antigen to T lymphocytes. Dendritic cells (DCs) are the most common and powerful APCs. The presentation of antigen affects through major histocompatibility complex protein (MHC). The DCs play crucial role in immunization and immunotolerance balance, which is strongly associated with cell maturation [50]. Immature dendritic cells stimulate regulatory (suppressor) T cell proliferation, whereas mature DCs, capable of antigen presenting, promote naive T cells to differentiate to Th1 lymphocytes and enhance pro-inflammatory response. 

Calcitriol, through several cytokines, e.g., anti-inflammatory IL-10, suppresses DCs maturation and MHCs type II expression and diminishes co-stimulating of antigens CD40, CD80, CD84. These processes increase the immunotolerance. The switch in interleukin production involves activation of nuclear factor kB (NF-kB) and mitogen activated protein kinase (MAPK) pathways in DCs. Subsequently, vitamin D_3_ inhibits pro-inflammatory interleukins (IL-12, TNF-α, INF-γ) and promotes T-cell inhibitory factors (programmed death-1, PD-1), decreasing activity and differentiation to Th1 and Th17 cells [51,52].

The T cell population differentiates into T helpers (Th, CD4+) and cytotoxic T cells (Tc, CD8+). Within the group of Th cells, there can be distinguished, among others, Th1, Th2, and Th17 cells. Maturation from naive T CD4+ to Th1 cells involves presenting the antigen by APCs in lymph nodes. VDR is present within the naive T cells, thus the vitamin D_3_ can directly influence T cell responses. Furthermore, calcitriol affects the differentiation of the T cell subclasses by inhibiting naive CD4+ T cells proliferation to Th1 cells and promoting maturation of Th2 cells. Moreover, vitamin D_3_ suppresses pro-inflammatory cytokine production (IL-2, INF-γ) by Th1 cells and simultaneously increases the production of anti-inflammatory cytokines by Th2 cells (IL-4, IL-5, IL-10) [53]. Therefore, vitamin D_3_ controls the Th1/Th2 immune balance and limits the Th1-induced destructive impact on tissues. Immune cells producing IL-17 (Th17) represent a quite new T cell subclass. The cytokine profile of Th17 cells (TNF-α, IL-6, IL-17, IL-21, IL-22) indicates that Th17 cells stimulate pro-inflammatory response in many diseases [54,55]. Probably, Th17 cells play a significant role in autoimmune diseases through pro-inflammatory cytokines, as there are many tissues that express receptors for IL-17 and IL-22. There have been published studies that provide the evidence of suppressive action of vitamin D_3_ on Th17 cells, leading to the decreased production of IL-17 and other cytokines (IL-1, IL-6, IL-12) and inhibition of CD4+ cells differentiation to Th17 [56]. Moreover, vitamin D_3_ stimulates IL-10 production by regulatory T cells (Treg) and proliferation of Treg, which subsequently regulate Th activation and cytokine production, affecting general immunological response [57]. 1,25(OH)_2_D_3_ is also speculated to affect B lymphocytes, but this relationship remains controversial. Vitamin D_3_ inhibits production of immunoglobulins (IgG and IgM), decreases the proliferation and maturation of memory B cells, and promotes the apoptosis of B cells [58]. Furthermore, B cells also express enzymes concerned in vitamin D_3_ metabolism (1α-hydroxylase and 24-hydroxylase), indicating a possible important role of vitamin D_3_ in B cell activity [53]. Unfortunately, mature B cells seem to be resistant to 1,25(OH)_2_D_3_ influence [58].

Vitamin D_3_ also affects the function of phagocytes (macrophages, monocytes). Vitamin D_3_ deficiency may manifest by diminished antimicrobial action of monocytes. 1,25(OH)_2_D_3_ decreases the expression of toll-like receptor (TLR2 and TLR4), decreases the production of pro-inflammatory molecules like TNF-α, but also stimulates monocytes to differentiate into macrophages, which take part in phagocytosis, chemotaxis and IL-1 production [59,60]. Moreover, vitamin D_3_ stimulates leukocytes and some epithelial cells (e.g., oral epithelial, intestines, vagina, keratinocytes) to produce antimicrobial proteins (defensin, cathelicidin). Antimicrobial proteins have strong antibacterial, antifungal and antiviral activity. Mechanisms of action include cell membrane destruction, suppression of microbe protein biosynthesis, and inhibition of nucleic acid biosynthesis or cell division [61].

## 5. Role of Vitamin D_3_ in Autoimmune Thyroid Diseases

Autoimmune thyroid diseases (AITD), including two main clinical manifestations: Hashimoto thyroiditis (HT) and Graves’ disease (GD), represent some of the most common autoimmune diseases, concerning about 5% of the population [12]. AITD are caused by autoimmunization to native antigens, e.g., thyroid stimulating hormone (TSH), thyroid peroxidase (TPO), thyroglobulin (Tg), and TSH-receptor (TSHR). Anti-TPO and anti-Tg are mostly connected with HT, whereas the anti-TSHR (TRAb) is related to GD.

HT is characterized by the lymphocytic infiltration and imbalance between Th1/Th2 lymphocytes [62]. This chronic inflammatory process leads to the destruction of thyroid follicles and is the most common cause of hypothyroidism in the iodine-sufficient population. Within the thyroid, activated Th1 lymphocytes produce TNF-α and INF-γ, which stimulate thyrocytes to secrete CXC10 (C-X-C motif chemokine ligand 10), responsible for the chemotaxis of monocytes, macrophages, T cells, and NK cells, and enhances the autoimmune vicious circle [13].

Having considered the fact that vitamin D_3_ plays immune-modulative effect, it may be speculated that vitamin D_3_ influences autoimmune inflammation in the thyroid gland in a particular way. Calcitriol affects antigen presenting cells, such as dendritic cells, decreases lymphocyte Th1 activation, and decreases the production of proinflammatory cytokines (IL-2, IL-12, TNF-α). Furthermore, vitamin D_3_ suppresses naive T cells differentiation into Th17 lymphocytes, inhibits Th17-connected interleukins secretion (IL-6, IL-17, IL-21, TNF-α), and thus promotes Treg activity and influences Treg/Th17 ratio. Moreover, vitamin D_3_ can influence the expression of MHC class II within the thyroid by its downregulation, and consequently limit thyroid autoantigens presentation by APCs to lymphocytes. Additionally, vitamin D_3_ decreases maturation and proliferation of B lymphocytes. Therefore, secretion of immunoglobulins IgG and IgM is limited [10,63].

GD is one of the most frequent form of hyperthyroidism in population. Although, the exact cause of GD is still unclear, it is assumed that particular environmental triggers activate proliferation of Treg cells, stimulate maturation of B cells to DCs, and lead to production of thyroid-antibodies. Autoantibodies in GD (TRAb) constantly stimulate TSH-receptors, and subsequently lead to proliferation of thyrocytes and synthesis of thyroid hormones (triiodothyronine, thyroxine), thyroid-specific proteins, and enzymes [64]. Vitamin D_3_ inhibits maturation of dendritic cells and inhibits secretion of inflammatory interleukins (IL-2, IL-12, IL-23, TNF-α, INF-γ). Furthermore, vitamin D_3_ modulates immune response by a direct influence on Th1 and Th2 lymphocytes, namely by down-regulating Th1 cells activity and up-regulating Th2 cells, as well as Th2-derived cytokines. As mentioned, vitamin D_3_ can also reduce proliferation of B lymphocytes and production of immunoglobulins that are associated with GD [10].

The relationship between vitamin D_3_ and AITD has been investigated in recent studies. Kivity et al. [65] noticed that vitamin D_3_ deficiency was statistically higher in patients with AITD in comparison to healthy individuals (72% versus 30.6%; *p* < 0.001). What is more, patients with hypothyroidism with AITD and no-AITD were also characterized with lower vitamin D_3_ levels (79% versus 52%; *p* < 0.05). Decreased concentrations of vitamin D_3_ appeared to be correlated with increased titer of antithyroid antibodies (*p* = 0.01). Bozkurt et al. [66] analyzed in their study in total 580 patients, including: newly diagnosed AITD, ongoing AITD and healthy volunteers. The author noticed that the concentration of vitamin 25(OH)D_3_ in patients with HT was significantly lower compared to the control group (*p* < 0.001) and the severity of vitamin D_3_ deficiency was corelated with the duration of HT and the titer of TPO and Tg antibodies (*p* < 0.001). Ma et al. [67] observed lower levels of vitamin D_3_ in patients with AITD (HT and GD) and each 5 nmol/L increase in serum 25(OH)D_3_ concentrations was associated with a 1.55- and 1.62-fold reduction in GD and HT morbidity. Botelho et al. [68] compared the group of 88 patients with HT with 71 healthy, euthyroid individuals and found an association between vitamin D_3_ deficiency and cytokines produced by Th1, Th2 and Th17 cells like TNF-α, IL-5 and IL-17 in patients with AITD. Fang et al. [69] confirmed a positive correlation between antithyroid antibodies, vitamin D_3_ deficiency (odds ratio (OR): 2.428, 95% confidence interval (CI): 1.383–4.261), and 25(OH)D_3_ inadequacy (OR: 1.198, 95% CO: 0.828–1.733; *p* = 0.008). The authors also found significantly higher quantities of Th1 and Th17 cells, as well as Th1 and Th17 associated cytokines in HT patients. Chao et al. [70] revealed that the level of 25(OH)D_3_ in the HT group was lower than in the non-HT group. The authors noticed a significant difference in thyroid function, namely that the thyroid-stimulating hormone (TSH) levels were significantly higher in both the 25(OH)D_3_ insufficiency group as well as the 25(OH)D_3_ deficiency group comparing to the 25(OH)D_3_ sufficiency group. In addition, the free triiodothyronine (FT_3_) and thyroxine (FT_4_) levels were significantly lower in the 25(OH)D_3_ insufficiency group. Moreover, the multiple regression analysis showed that HT was significantly correlated with male sex, body mass index (BMI), waist circumference, and TSH [70].

Within the literature, there can also be found a few studies which did not confirm the relationship between AITD and the vitamin D3 deficits. Effraimidis et al. [71] examined 156 participants and did not find association between low vitamin D_3_ level and AITD. D’Aurizio et al. [72] analysed 100 patients affected AITD (both HT and GD) and 126 healthy subjects. The authors did not find a significant correlation between vitamin D_3_ levels and presence of AITD. Ke et al. [73] observed that, in patients diagnosed with AITD, serum 25(OH)D_3_ levels were not associated with thyroid function, presence of antithyroid antibodies, or serum cytokines IL-4, IL-17, and TNF-α. The authors found moderately lower vitamin D_3_ levels in HT patients, whereas in GD patients the levels of 25(OH)D_3_ were comparable to the values presented within the control group. Ma et al. [67] did not find any significant relationship between serum 25(OH)D_3_ level or any of the below listed: titer of anti-TPO, anti-TG, or TSH serum level [67].

Although, there have been mentioned a few manuscripts which did not confirm the relationship between vitamin D_3_ and AITD, there are still a lot of publications which state that the relationship between vitamin D_3_ with AITD remains indisputable. Further studies are needed to thoroughly evaluate the clinical effects of vitamin D_3_ on AITD [74].

Table 1 presents the relationship between vitamin D_3_ and AITD on the basis of the literature [65,66,67,68,69,70,71,73].

## 6. Vitamin D_3_ and Bone Metabolism

Vitamin D_3_ plays a prominent role in calcium-phosphate and bone metabolism. The overall feature of vitamin D_3_ is to increase and maintain the accurate concertation of calcium and phosphate within the extracellular fluid (ECF). Thus, it creates adequate conditions for proper growth, bone remodeling, as well as mineralization of skeleton [3]. Health bone homeostasis is controlled by the osteoblasts (bone-forming mesenchymal-derived cells) and osteoclasts (bone resorption, multi-nucleus hematopoietic stem-derived cells) [75].

Calcitriol increases the concentrations of calcium and phosphates in ECF through intensified intestinal absorption from nutrients and renal reabsorption from primary urine in proximal tubule. Moreover, it increases the expression of transmembrane glycoprotein Receptor Activator for Nuclear Factor κβ Ligand (RANKL) on osteoblasts. RANKL binds to Receptor Activator for Nuclear Factor κβ (RANK) on preosteoclasts and stimulates their differentiation to mature osteoclasts and accelerates bone resorption [76]. Secondly, 1,25(OH)_2_D_3_ stimulates osteoblasts to produce proteins essential in the processes of bone remodeling and mineralization of bone matrix, namely collagen, osteopontin, osteocalcin (dependent on vitamin K bone protein). Moreover, 1,25(OH)_2_D_3_ stimulates the activity of alkaline phosphatase, which is essential in the process of bone mineralization [77]. Furthermore, vitamin D_3_ promotes bone forming by accelerating the differentiation of monocytes to macrophages, as well as through binding them to the osteoclasts, which enhances bone resorption and calcium release from the bones [78]. Moreover, 1,25(OH)_2_D_3_ also plays a significant role in the upregulation of osteoprotegerin (OPG), a soluble glycoprotein, which is a competitive RANKL inhibitor. OPG after being bound to RANKL, inhibits the activation of RANK by its ligand, and subsequently inhibits the processes of osteoclasts activity, maturation and differentiation [79]. Thus, the vitamin D_3_ apparently affects bone development, mineralization, and remodeling through its resorption.

Parathormone (PTH) is the one of the most important hormones in bone metabolism. PTH is produced in parathyroid glands. Parathyroid cells, along with renal tubules, brain, heart, skin, stomach, and C cells, express calcium sensing receptors (CaSR), a Class III or Family C G-protein coupled receptor [80]. Serum ionized calcium binds to CaSR and transduces signals through phospholipase C, which hydrolyses phosphatidylinositol 4,5-bisphosphonate to diacyl glycerol (DAG) and inositol 1,4,5-triphosphate (IP3). IP3/DAG pathway leads to the degranulation of calcium in endoplasmic reticulum, increased intracellular calcium concentration, and finally blocks degranulation of the vesicles with PTH to the cell membrane. Thus, the secretion of PTH through parathyroids is inhibited [81].

Parathormone activity is strictly corelated with 1,25(OH)_2_D_3_ by negative feedback. Increased concentration of calcitriol inhibits CaSR and decreases PTH serum level. Parathormone plays dual role: both anabolic (bone remodeling), and catabolic (bone resorption) ones. On the one hand, pulsating secretion of PTH, through parathyroid receptor type 1 (which belongs to G protein-coupled receptor family), stimulates osteoblasts to produce important compounds for bone matrix composition, namely insulin-like growth factor 1 (IGF-1), fibroblast growth factor (FGF), matrix metalloproteinase (MM-13), or Wnt/β-catenin [82]. Moreover, PTH decreases osteoblasts’ apoptosis and intensifies bone matrix formation [83]. On the other hand, PTH indirectly leads to bone resorption through the activation of osteoclasts. PTH downregulates the production of osteoprotegerin, promotes binding RANKL to RANK, and stimulates the differentiation of osteoclasts. Having been activated, osteoclasts produce hydrogen ions, via carbonic anhydrase, to dissolve mineralized matrix into water and ions: calcium, magnesium, phosphates, and other organic substances. Simultaneously, particular hydrolytic enzymes, including cathepsin K and MM-13, are secreted to degrade proteins from bone matrix [84]. During the process of bone resorption, calcium, magnesium and phosphate ions are released to ECF and subsequently to blood circulation. Parathormone also affects kidneys. As mentioned before, it increases the activity of 1α-hydroxylase, and therefore 1,25(OH)_2_D_3_ is produced. Furthermore, PTH increases the reabsorption of calcium in renal proximal tubule [85]. To sum up, PTH controls the calcium homeostasis within three stages, namely: bone resorption, vitamin D_3_ activation, and both intestine and renal calcium absorption.

## 7. Vitamin D_3_ and Osteoarthritis

Osteoarthritis (OA) is a chronic, degenerative disease involving joint cartilage, synovium, periarticular ligaments, and subchondral bone, affecting up to one in eight adults. It is the most common chronic articular disease. Only a few effective methods of OA treatment have been discussed, but none of them is able to stop or effectively delay the development of the disease [86]. 

The constantly increasing number of patients diagnosed with OA is associated with increasing life expectancy of population. The cause of the disease is multifactorial, including modifiable and non-modifiable, local and systemic factors. Among the risk factors, there have been mentioned: female sex, race, genetics, old age, type of diet, overweight and obesity, joint injury and mechanical factors, repetitive use of joints, bone density, muscle weakness and joint laxity, low-grade-inflammatory processes, and hormonal system [87,88]. OA can be characterized pathologically, radiographically, and clinically. Diagnosis of OA is not always evident, because some patients with radiological symptoms do not present any clinical manifestation and, at the same time, not everyone with joint symptoms present radiological changes. Therefore, OA should be diagnosed with a diversity of methods, including pathological, clinical, and radiological [89].

The articular cartilage is made of water (>70%) and organic matrix, mostly type II collagen, aggrecan and other proteoglycans [90]. The pathophysiological background of OA involves the whole group of pro-inflammatory cytokines (interleukins IL-1β, IL-6, IL-8), as well as pro-catabolic signalization with nuclear factor kB (NF-kB), mitogen activated protein kinase (MAPK) pathways, and the activation of synovial macrophages and fibroblasts [91]. The inflammatory stimulation of chondrocytes results in upregulation of proteinases, especially aggrecanase and collagenase. The main enzymes responsible for degradation of cartilage matrix are A Disintegrin and Metalloproteinase with Thrombospondin motifs (ADAMTS) and zinc-dependent metalloproteinases (MMPs) belonging to the MMP families. The MMPs group includes collagenases MMP-1, MMP-13 (type II collagen proteinase), and MMP-3 (effective aggrecanase) [92]. The additive effect of pro-inflammatory mediators, mechanical injuries, and oxidative stress affect the function and vitality of chondrocytes, resulting in further degeneration of cartilage and bone underneath.

Recent studies suggest that vitamin D_3_ may play a significant role in osteoarthritis. Several studies revealed that chondrocytes express VDR. Orfanidou et al. [93] showed increased expression of VDR in the areas of cartilage erosion in OA. In a prospective study with 418 participants with already diagnosed OA Zhang et al. [94] found that participants with both decreased concentration vitamin D_3_ and high concentration of PTH had a more than three-fold increased risk of OA progression. Heidari et al. [95] presented similar conclusions, however the significant difference was observed in the younger group of patients (<55 years, *p* = 0.01), whereas in patients aged more than 60 years old the association between serum 25(OH)D_3_ deficiency and OA was not statistically significant. In another, two-year prospective study with 413 enrolled participants with knee OA and low 25(OH)D_3_ serum level, Jin X et al. [96] revealed that treatment with 50 000 IU of vitamin D_3_ monthly did not result in significant changes in MRI-measured tibial cartilage volume or WOMAC (Western Ontario and McMaster Universities Osteoarthritis Index) knee pain score. Contrary to this, Gao XR et al. [97] found that daily supplementation of more than 2000 IU of vitamin D_3_ significantly decreased pain and improved function of the joint on the basis of the WOMAC scale. However, the authors did not find any beneficial effect of vitamin D_3_ supplementation on the prevention of tibial cartilage loss. What is more, Divjak et al. [98] administrated 4000 IU of 25(OH)D_3_ daily to patients with primary knee OA and compared the cytokine profile before and after intervention. The authors observed that as the concentration of IL-1β (*p* < 0.01), IL-23 (*p* < 0.01), and IL-33 (*p* < 0.01) significantly increased, the concentration of TNF-α (*p* < 0.01), IL-13 (*p* < 0.01), and IL-17 (*p* < 0.01) significantly decreased, whereas the concentration of IL-4 did not change significantly. The prescribed treatment with vitamin D_3_ appeared to reduce joint pain, joint stiffness, and to improve physical function. The authors suggested that vitamin D_3_ supplementation may be recommended as a new co-therapeutic treatment in the course of knee OA.

Vitamin D_3_ is also known to affect bone regeneration, bone malformation, as well as osseointegration of implants. There have been published several metanalyses and systematic reviews regarding the relationship between vitamin D_3_ and abovementioned processes [99,100,101]. Salomó-Coll et al. [102] performed an animal study and noticed reduced crestal bone loss as well as increased osteointegration by 10% around implants supplemented with vitamin D. Dvorak et al. [103] described that vitamin D deficiency negatively affected implants osseointegration in rats. Werny et al. [100] stated that 75% of the analyzed studies had confirmed the positive effect of vitamin D supplementation on bone regeneration. Unfortunately, most of the abovementioned studies were performed on animals. So far, several studies have been performed on human patients, but the results are often contradictory. Kwiatek et al. [104] showed, in a prospective, randomized clinical trial on a group of 122 patients, that vitamin D supplementation and treatment of vitamin D deficiency led to an increased bone level surrounding the implant 12 weeks after surgery. Contrary to this study, Grønborg et al. [105] performed a randomized double-blinded placebo-controlled trial with 143 women diagnosed with vitamin D deficiency who received special diet reach in vitamin D. After 12 weeks the author noticed a statistically significant increase in vitamin D serum concentration (*p* < 0.05). However, they did not notice significant changes in bone turnover biomarkers, nor improvement in muscle strength. Having considered all of the above mentioned, further randomized human studies are necessary to establish the role of vitamin D_3_ in the process of implant osseointegration.

Undoubtedly, vitamin D_3_ affects the metabolism of bone and chondrocytes by the modulation of pro- and anti-inflammatory responses. However, there are no specific guidelines regarding the use of vitamin D_3_ in the treatment of OA. In light of the latest research, the use of vitamin D_3_ in patients diagnosed with OA seems to be promising.

Table 2 presents the relationship between vitamin D_3_ and OA on the basis of the literature [93,94,95,96,98].

## 8. Vitamin D_3_ and Temporomandibular Joint Osteoarthritis

Temporomandibular disorders (TMD) is an umbrella term describing the pathology within the temporomandibular joints and/or adjacent muscles [106]. The most typical symptoms for TMD include: pain in the area of TMJs, limited mouth opening, and noises within the TMJs [106]. The etiology of TMD is multifactorial and encompasses several different items, which may be allocated into one of the subgroups, namely: host-adaptive capacity factors (genetics, age, estrogens, systemic diseases, abnormal remodeling of subchondral bone) and mechanical factors (excessive mechanical stress, parafunctions, functional overloading, microtrauma) [107,108]. In the past, it was believed that occlusion played a major role in the development of TMD [109,110]. However, according to the most recent research, there is not enough evidence to confirm that occlusion may lead to TMD [111,112,113]. TMD is a complex condition, modified by many factors, and which often binds with many diseases [114,115].

Because of the fact that TMD is not a dental, but an interdisciplinary issue, the treatment of TMD, including temporomandibular joint osteoarthritis (TMJ OA), requires the cooperation of different specialists, including physiotherapists, dentists, rheumatologists, endocrinologists, laryngologists, maxillofacial surgeons, psychiatrists, psychologists, and speech therapists.

TMJ OA is a degenerative joint disease (DJD). According to the Diagnostic Criteria for Temporomandibular Disorders (DC/TMD), to diagnose DJD, it is necessary to meet the following criteria: noises within the TMJ that occur during the movement of the mandible within the last 30 days or noise within the TMJ which is reported by the patient during the examination [116]. Moreover, it is necessary to detect the crepitus with palpation during mandibular movement [116]. Nonetheless, the diagnosis based only on anamnesis and clinical examination, without imaging, is characterized by sensitivity of 0.55 and specificity of 0.61 [116]. Radiological symptoms typical for DJD are: erosion, osteophytes, generalized sclerosis, and subchondral cysts. According to the DC/TMD, articular surface flattening and cortical sclerosis are regarded as indeterminant findings for DJD [116].

As previously stated, vitamin D_3_ plays a significant role in calcium-phosphate and bone metabolism [3,75]. It was also reported that vitamin D_3_ deficits may be correlated with the development and progression of osteoarthritis [93,94]. Although it may be speculated that low serum concentration of vitamin D_3_ is correlated with the development and/or progression of TMJ OA, so far there have been published only few studies regarding this topic and the results remain inconsistent.

Shen et al. [117] examined 25(OH)D_3_ 1α-hydroxylase knockout mice which had been fed a rescue diet. The authors found that 1,25(OH)_2_D_3_ deficient mice presented reduced bone mineral density and reduced subchondral bone volume within the mandibular condyles. Shen et al. [117] also reported that 1,25(OH)_2_D_3_ deficiency was associated with the changes in the shape of articular surfaces, as well as in the thickness of articular cartilage. The authors observed articular surface erosion in 1,25(OH)_2_D_3_ deficient mice. Shen et al. [117] concluded that 1,25(OH)_2_D_3_ deficiency was correlated with erosive TMJ OA phenotype, increased DNA damage, cellular senescence, as well as with the production of inflammatory cytokines associated with senescence.

Hong et al. [118] noticed that 1,25(OH)_2_D_3_ was significantly correlated with TMJ OA, both development and progression, in young and postmenopausal women. The authors indicated that vitamin D_3_ could be considered a therapeutic agent for TMJ OA. Jagur et al. [119] indicated that decreasing bone mineral density as well as low serum concentration of 25(OH)D_3_ may be considered predictors of bone destruction in the area of TMJs. Gupta et al. [120] found that in patients diagnosed with TMD, who were also vitamin D_3_ deficient, supplementation of vitamin D_3_ in addition to stabilization splint therapy led to quicker alleviation of pain in the area of TMJs. Demir et al. [121] compared healthy individuals and patients with TMD. The authors did not find statistically significant differences between the examined groups regarding the serum concentration of: calcium, magnesium, phosphorus, calcitonin, and 25(OH)D_3_. Patients diagnosed with TMD presented significantly increased concentration of parathyroid hormone. According to Demir et al. [121], vitamin D_3_ deficiency in patients diagnosed with TMD requires assessment and correction. Madani et al. [122] performed a case-control study and found that there were no statistically significant differences in the serum concentrations of: alkaline phosphatase, phosphate, calcium, PTH, and vitamin D_3_ between patients diagnosed with TMD and healthy individuals.

To sum up, it may be speculated that vitamin D_3_ concentrations may be different in various TMDs, which may consequently be the cause of inconsistency in the obtained results from the above presented research. Studies in which only cases with TMJ OA were included clearly indicated that 1,25(OH)_2_D_3_ was significantly correlated with TMJ OA [110,111]. Kui et al. [123] concluded that further studies regarding the relationship between vitamin D_3_ concentration and TMDs are absolutely needed and that supplementation of vitamin D_3_ is recommended for vitamin D_3_ deficient patients who suffer from TMD.

## 9. Conclusions

Calcitriol impresses a pleiotropic effect on the human biology and metabolism. Its modulative function upon the immune system is based on the reduction of Th1 cell activity and increased immunotolerance. Severe vitamin D_3_ deficiency may lead to an imbalance in relationship between Th1/Th17 and Th2, Th17/Th reg, and is considered by some authors as one of the possible backgrounds of autoimmune thyroid diseases (AITD), e.g., Hashimoto’s thyroiditis or Graves’ disease. Moreover, vitamin D_3_, through a direct and indirect influence on bones and joints, may also play an important role in the development and progression of degenerative joint diseases, including temporomandibular joint osteoarthritis. Further randomized, double blind studies are needed to unequivocally confirm the relationship between vitamin D_3_ and abovementioned diseases and to answer the question concerning whether vitamin D_3_ supplementation may be used in the prevention and/or treatment of either AITD or OA diseases.

## Figures and Tables

**Table 1:** The relationship between vitamin D_3_ and AITD on the basis of the literature [65,66,67,68,69,70,71,73].

Reference	Study Design	Participants and Intervention	Results
Kivity et al. (2021) [65]	Case control study	92 patients (71 women, 21 men, mean age—group AITD: 45 ± 16 years; group non-AITD: 52 ± 16 years): - AITD (50 patients) - non-AITD (42 patients) - control group: healthy participants (98 patients) - blood tests were taken in all of the participants	The prevalence of vitamin D_3_ deficiency was: - significantly higher in patients with AITD compared to healthy individuals (72% versus 30.6%; *p* < 0.001), - in patients with HT compared to patients with non-AITDs (79% versus 52%; *p* < 0.05) - correlated to the presence of antithyroid antibodies (*p* = 0.01)
Bozkurt et al. (2013) [66]	Case control study	560 patients: - group 1: euthyroid patients with ongoing HT (180 patients: 123 women, 57 men) - group 2: euthyroid subjects with newly diagnosed HT (180 patients, sex-, age-, and BMI-matched) - control group: healthy volunteers (180 patients) - blood tests were taken in all of the participants	Group 1 presented the lowest concentrations of vitamin D_3_ (11.4 ± 5.2 ng/mL) having compared to newly diagnosed HT subjects (Group 2) (13.1 ± 5.9 ng/mL, *p* = 0.002), as well as to control subjects (15.4 ± 6.8 ng/mL, *p* < 0.001) Serum vitamin D_3_ concentrations correlated positively with thyroid volume (r = 0.145, *p* < 0.001) and negatively with anti-TPO (r = −0.361, *p* < 0.001), as well as with anti-Tg levels (r = −0.335, *p* < 0.001).
Ma et al. (2015) [67]	Case control study	210 patients: - GD (70 patients: 48 women, 22 men, mean age: 40.04 ± 15.24 years) - HT (70 patients: 51 women, 19 men, mean age: 40.11 ± 14.60 years) - Control (70 patients: 49 women, 21 men, mean age: 41.99 ± 13.31 years) - blood tests were taken in all of the participants	AITD patients presented significantly lower levels of 25(OH)D3 comparing to the control group (*p* < 0.001). Every 5 nmol/L increase in serum 25(OH)D3 concentrations led to reduction in GD and HT morbidity by 1.55-, and 1.62-fold. There were no significant relationships between serum 25(OH)D3 concentration and any of the below listed: titer of anti-TPO, anti-Tg, and TSH serum level.
Botelho et al. (2018) [68]	Case control study	159 patients: - HT (88 patients: 82 women, 6 men, mean age: 42.2 years, age range: 20–66 years) - euthyroid healthy subjects (71 patients: 61 women, 10 men, mean age: 47.4, age range: 19–77 years) - blood tests were taken in all of the participants	Vitamin D3 concentrations did not differ significantly between HT patients and the control group (*p* = 0.1024). In HT group there were found positive correlations between vitamin D3 concentration and the below listed: free T4 (*p* = 0.0224), TNF-α (*p* = 0.0004), IL-5 (*p* = 0.0144), IL-17 (*p* = 0.0011).
Fang et al. (2021) [69]	Case control study	1812 patients: - anti-TPO positive (237 patients: 171 women, 66 men, mean age: 45.76 ± 15.06 years) - anti-TPO negative (1575 patients: 880 women, 695 men, mean age: 42.39 ± 14.97 years) - anti Tg positive (254 patients: 196 women, 58 men, mean age: 45.11 ± 15.09 years) - anti Tg negative (1558 patients: 855 women, 703 men, mean age: 42.46 ± 14.98 years) - blood tests were taken in all of the participants	Vitamin D3 deficiency was associated with increased likelihood of positive anti-TPO ([OR]: 2.428, 95%, [CI]: 1.383–4.261), as well as with positive anti-TG was (OR: 2.366, 95% CI: 1.366–4.099). HT patients, compared to healthy group, presented a significantly higher proportions of Th1 and Th17 cells, as well as significantly higher level of related cytokines.
Chao et al. (2020) [70]	Case control study	5230 patients: - non-HT (4889 patients: 1851 women, 3038 men, mean age: 48.99 ± 9.04 years) - HT (373 patients: 248 women, 125 men, mean age: 48.51 ± 9.36 years) - blood tests were taken in all of the participants	The concentration of vitamin D3 was significantly lower in the HT group comparing to the non-HT group (*p* = 0.014). 25(OH)D3 deficiency and insufficiency groups presented significantly higher concentrations of TSH comparing to the 25(OH)D3 sufficiency group (*p* < 0.001). The concentrations of FT3 and FT4 were significantly lower in the 25(OH)D3 deficiency and insufficiency groups (*p* < 0.001) comparing to 25(OH)D3 sufficiency group.
Effraimidis et al. (2012) [71]	Two case control studies	Study A: 156 patients (156 women): - healthy euthyroid thyroid antibody-1 negative female relatives of AITD patients (78 women, mean age: 42.1 ± 13.2 years) - healthy controls (78 women, mean age: 42.3 ± 13.1 years) Study B: - healthy euthyroid thyroid antibodies-negative women who developed anti-TPO during 5-year follow-up (67 women, mean age: 38.3 ± 11.5 years) - healthy women who did not develop anti-TPO during 5-year follow-up (67 women, mean age: 38.1 ± 11.3 years) - blood tests were taken in all of the participants in both studies	Healthy euthyroid thyroid antibody-1 negative female relatives of AITD patients presented significantly higher serum 25(OH)D3 concentration compared to the control group (*p* = 0.01). The prevalence of 25(OH)D3 deficiency (<20 ng/mL) occurred less often in female relatives of AITD patients comparing to healthy controls (*p* = 0.05). The concentrations of the 1,25(OH)2D3 and 25(OH)D3 did not differ significantly between patients who developed anti-TPO and healthy controls. There was no association between low concentration of vitamin D3 and early stages of thyroid autoimmunity.
Ke et al. (2017) [73]	Case control study	226 patients: - patients diagnosed with AITD (175 patients) * GD (51 patients, 30 women, 21 men, mean age 39.79 ± 1.73) * euthyroid HT, mild HT (61 patients, 34 women, 27 men, mean age 40.88 ± 1.61) * euthyroid HT patients with hypothyroidism receiving, treated HT (63 patients, 35 women, 28 men, mean age 42.41 ± 1.49) - healthy controls (51 patients, 31 women, 20 men, mean age 36.48 ± 1.68) - blood tests were taken in all of the participants	Patients from groups: treated HT and mild HT presented significantly lower vitamin D3 levels (*p* < 0.001) comparing to the controls. The concentration of vitamin D3 did not differ significantly between GD patients and healthy controls. Within the AITD group there were no correlations between vitamin D3 serum levels and thyroid hormones, antithyroid antibodies, as well as serum cytokines TNF-α, IL-4 and IL-17.

25(OH)D_3_—25-hydroxyvitamin D_3_, AITD—autoimmune thyroid diseases, BMI—body mass index, FT_3_—free triiodothyronine, FT_4_—free thyroxine, GD—Graves’ disease, HT—Hashimoto’s thyroiditis, IL-4—interleukin 4, IL-5—interleukin 5, IL-17—interleukin 17, non-AITD—non-autoimmune thyroid diseases, T_4_—thyroxine, Tg—thyroglobulin, TNFα—tumor necrosis factor α, TPO—thyroid peroxidase, TSH—thyroid-stimulating hormone.

**Table 2:** The relationship between vitamin D_3_ and OA on the basis of the literature [93,94,95,96,98].

Reference	Study Design	Participants and Intervention	Results
Orfanidou et al. (2012) [93]	Case control study	50 patients: - patients with end-stage primary OA undergoing knee replacement surgery (40 patients, 30 women, 10 men, mean age: 64.18 + 14.24 years) - control group, healthy cartilage samples obtained during fracture repair surgery (10 patients, 7 women, 3 men, mean age: 44.60 + 7.6) - blood tests were taken in all of the participants	The expression levels of FGF23, FGFR1c, VDR, PiT-1, and PiT-2 were significantly higher in patients diagnosed with OA comparing to the control group.
Zhang et al. (2014) [94]	Case control study	418 patients: - patients with at least one knee with radiographic symptoms of OA and frequent pain/aching or stiffness in the area of knee (418 patients, 195 women, 223 men, mean age: 61.0 ± 9.2) - blood tests were taken in all of the participants	Low serum vitamin D_3_ concentration was associated with an increased risk of OA progression.
Heidari et al. (2011) [95]	Case control study	298 patients: - knee OA group (148 patients, mean age: 60.2 ± 12.9 years) - control group (150 patients, mean age: 60.1 ± 10.2 years) - blood tests were taken in all of the participants	Mean serum vitamin D_3_ concentration did not differ significantly bwteen OA patients and the control group (*p* = 0.28). Mean serum vitamin D_3_ in OA patients aged below 60 years old was significantly lower comparing to the control group (*p* = 0.01). In patients younger than 60 years old the knee OA was significantly correlated with vitamin D_3_ deficiency (*p* = 0.018).
Jin et al. (2016) [96]	Randomized clinical trial	413 patients with symptomatic knee OA for at least 6 months: - oral vitamin D_3_ group (209 patients, 106 women, 103 men, mean age: 63.5 + 6.9 years)—monthly intake of 50 000 IU of vitamin D_3_ for 24 months - placebo group (204 patients, 102 women, 102 men, mean age: 62.9 + 7.2 years) - all patients underwent MRI examination of the knee - blood tests were taken in all of the participants	The serum level of 25(OH)D3 increased significantly more in the vitamin D3 group comparing to the placebo (*p* < 0.001). Neither tibial cartilage volume nor WOMAC pain score changed significantly. Supplementation of vitamin D3 does not prevent tibial cartilage loss nor improves WOMAC knee pain.
Divjak et al. (2022) [98]	Randomized clinical trial	80 patients diagnosed with knee OA: - oral vitamin D_3_ solution group (60 patients, 35 women, 25 men, mean age: 57.4 + 4.2 years)— 4 000 IU of vitamin D_3_ per day for 3 months - control group—without supplementation (20 patients, 12 women, 8 men, mean age: 56.1 + 4.3 years) - blood tests were taken in all of the participants	Vitamin D_3_ supplementation led to a significant pain reduction, both in VAS and WOMAC pain scores. The serum concentrations of IL-1β (*p* < 0.01), IL-23 (*p* < 0.01), and IL-33 (*p* < 0.01) significantly increased in patients who received vitamin D_3_ supplementation comparing to control group. The serum concentrations of IL-13 (*p* < 0.01), IL-17 (*p* < 0.01) and TNF-α (*p* < 0.01) significantly decreased in patients who received vitamin D_3_ supplementation comparing to control group.

25(OH)D_3_—25-hydroxyvitamin D_3_, FGF23—fibroblast growth factor 23, FGFR1c—fibroblast growth factor receptor 1c, IL-1β—Interleukin 1 beta, IL-13—interleukin 13, IL-17—interleukin 17, IL-23—interleukin 23, IL-33—interleukin 33, IU—international unit, OA—osteoarthritis, PiT-1—phosphate inorganic transporter-1, PiT-2—phosphate inorganic transporter-2, TNFα—tumor necrosis factor α, VDR—vitamin D_3_ receptor, WOMAC—Western Ontario and McMaster Universities Arthritis Index.

## Data Availability

The data underlying this article are available in the article.
